# Assessing the foundations of forensic identification evidence: A critical examination of proficiency test design and results

**DOI:** 10.1073/pnas.2528192123

**Published:** 2026-06-26

**Authors:** Nicholas Scurich, Thomas D. Albright

**Affiliations:** ^a^ Department of Psychology, School of Social Ecology, University of California, Irvine, CA 92697-7050; ^b^ Department of Criminology, Law & Society, School of Social Ecology, University of California, Irvine, CA 92697-7050; ^c^ Salk Institute for Biological Studies, La Jolla, CA 92037

**Keywords:** forensic science, decision making, expert evidence

## Abstract

Proficiency testing is widely used to assess expertise in medicine, engineering, and other high-stakes fields. In forensic science, such tests are often cited in court as evidence that pattern-comparison methods are accurate and reliable. Yet, the scientific value of proficiency testing depends on test design, scoring rules, and the extent to which results support valid inferences about real-world performance. A recent proficiency test of forensic firearm identification—the comparison of bullets and cartridge cases to determine whether they were fired from a particular gun—reported a false-positive error rate of nearly 20%. The broader significance of that result extends beyond the reported figure. We identify several limitations that can arise in forensic proficiency testing: use of items that may not reflect casework difficulty, reliance on consensus scoring rather than independently verified ground truth, inconsistent treatment of inconclusive responses, variation in test materials across examinees, and procedures vulnerable to contextual bias, including nonblind verification. These features make it difficult to distinguish examiner performance from properties of the test itself and weaken claims that reported scores estimate operational error rates. Properly constructed tests could measure individual differences in performance, identify sources of error, evaluate decision thresholds, and provide courts with more meaningful evidence about reliability. Although our focus is on firearms, these challenges extend to other pattern-comparison disciplines, including fingerprints and handwriting. We conclude that comprehensive, evidence-based reform of forensic proficiency testing is warranted.

Broadly considered, the human visual system performs two important functions: measurement and classification. This system enables us to assess physical attributes of the input, such as the size, shape, distance, brightness, and texture of visible objects. We use these measurements to classify objects based on similarity to things we have seen before.

Most visual classification decisions are categorical: It is a porcupine, or a five-dollar bill. But a subset are decisions about object identity: This is my car. That is the Koh-I-Noor diamond. Because of this facility, the human visual system has been employed for decades as a tool for forensic source identity decisions based on visually patterned evidence from a crime scene, such as fingerprints or markings on bullets and cartridges from a firearm.^*^

A common problem with identity claims is that they can be contentious. They assert ownership or causality over matters on which stakeholders may have a significant interest or may suffer irreparable harm. In forensics, an assertion of identity between pattern evidence from a crime scene and that from a hypothesized source is often tantamount to linking the crime scene item to a suspect and, in turn, suggesting criminal liability. Another problem with visual pattern identity claims is that they are commonly based on conjunctions of visual attributes measured and interpreted in unfamiliar contexts, rife with sources of noise and opportunities for bias. Moreover, a given observer’s visual similarity measurements and decision criteria for pattern identity are inscrutable to those receiving the classification.

This uncertainty and potential for bias present a dilemma for criminal courts that rely on forensic identity claims. To address this problem, a judicial decision about whether a forensic examiner was qualified to offer an opinion about identity in a court of law rested for many years upon the examiner’s “credentials,” including training and years of practice, which were assumed to be predictive of accuracy. As with other disciplines that require high-stakes decisions rendered under conditions of uncertainty, such as medicine, it is now widely accepted that credentials alone are not a satisfactory predictor of performance. Indeed, one cannot objectively assess the veracity of forensic identity claims based on a specific pattern comparison method without empirical validation (1, 2).

Recent years have seen numerous attempts to validate forensic pattern comparison disciplines. In the ideal, forensic validation studies blindly assign examiners to conditions for which the correct responses are known, to determine the probability that examiners’ decisions are correct. We focus here on firearms analysis, in which trained observers examine striations and indentations found on spent cartridge cases or bullets recovered from a crime scene and compare them with markings on materials from a known firearm. There have been a handful of large-scale validation studies on firearms analysis in recent years that report—uniformly and to a fault—that error rates for identity claims are very low (<1%) (3). These statistics have been used to justify this form of evidence in criminal trials and have been presented to jurors. Several independent analyses, however, have raised serious concerns about the quality of the data and fitness of the interpretation (4).

## Forensic Validation Through Proficiency Testing

A different approach to validation comes in the form of proficiency testing of forensic examiners. Unlike large-scale validation studies that seek to ascertain how well a technique generally performs, proficiency testing serves three related functions.^†^ First, by design, they are conducted to improve quality control within forensic laboratories, which includes assessing how well examiners conform to policy and procedure and identifying personnel who do not meet a minimum level of competence in performing casework when tested (5). In this capacity, proficiency testing functions like calibration checks on a measurement instrument. Passing calibration does not guarantee accurate field measurements, but failing calibration invalidates any claim of accuracy. Proficiency tests can be valuable because they reveal how methods perform when embedded in real institutional practice. In this sense, proficiency tests function as stress tests, exposing vulnerabilities that may be obscured in formal research.

Second, regular proficiency testing of laboratory personnel is required to achieve and maintain accreditation by professional organizations, such as the American Society of Crime Laboratory Directors/Laboratory Accreditation Board (ASCLD/LAB). This ensures and advertises a standard level of performance. Third, in addition to their use to enhance laboratory operations and accreditation, forensic proficiency tests are frequently employed de facto—although not explicitly designed for this purpose—as evidence of an examiner’s qualifications for an expert opinion proffered in a legal proceeding. In this latter role, proficiency tests have the potential to validate the error rate associated with identification claims, which would be of great value to the courts. As we shall see, however, there are numerous hurdles associated with reaching this potential.

As testament to the increasing use and potential value of findings from forensic proficiency tests, a commercial market has emerged for test administration and scoring. Collaborative Testing Services, Inc. (CTS) is a major provider (6). CTS publishes the results and conclusions of firearms proficiency tests for both expended cartridges and bullets at regular intervals on a publicly accessible site (7). These reports list individual examiner responses—“Yes” (same source as the sample), “No” (different source from the sample), or “Inconclusive”—to four questioned cartridges or bullets, with each response coded as correct or incorrect. Responses are aggregated to yield the frequency of each response type for each questioned item, from which accuracy (the probability of a correct decision) and its complement, the error rate, are inferred.

Generally, error rates on CTS firearms proficiency tests are remarkably low—1% or less (8). Examiners in turn boast about their flawless performance, claiming they have never made an error during these tests. This is not entirely surprising. CTS tests “are typically designed to be relatively easy for a competent analyst to pass,” and the President of CTS acknowledged that “he has been under commercial pressure to make proficiency tests easier” (9). Though this suggests that CTS proficiency tests are not a challenging assessment of ability, CTS error rates are frequently cited in court as evidence of a firearm examiner’s qualifications to render expert opinions (10). While CTS explicitly states that their tests do not establish industry-wide error rates, the reported rates are often discussed and implicitly treated as such during legal proceedings (11).

In 2023, however, CTS published firearm test results that shocked the forensic community (12). This was a proficiency test comparing fired bullets. The false-positive error rate for the group of examiners who submitted responses in this test was an alarming 20%. The Association of Firearm and Toolmark Examiners (AFTE), the leading professional organization of forensic firearms examiners, attributed these errors to examiner decision-making. In contrast, we argue that they point to deeper structural issues within the discipline and show how proficiency testing, when properly designed and administered, can reveal both strengths and weaknesses of a forensic technique.

## The 2023 CTS Test (Test 23-5262)

As with all other CTS bullet tests, examiners were given five items to compare in a match-to-sample paradigm. Item 1—the “sample”—was a “known” bullet, meaning the firearm that fired it was identified. Three exemplars of Item 1 were included in the test kits to facilitate comparison. Examiners were tasked with determining whether any of the other four bullets—Items 2, 3, 4, and 5—were fired by the gun that produced Item 1. Items 2, 3, and 5 were all fired by the same gun, but it was not the gun that fired Item 1. Item 4 was an out-of-class comparison, meaning it was fired by a completely different *type* of firearm and was therefore an “easy” elimination (13). In short, none of the four questioned bullets (Items 2, 3, 4, and 5) were fired by the same gun as Item 1.

Table 1 contains the frequency of response types (Yes, No, Inconclusive) for each of the four questioned items (Items 2 to 5). Because none of these items came from the same source as the sample (Item 1), all Yes responses are false-positive errors. If we exclude the results for item 4, which was an easy out-of-class comparison, the false-positive rate for the remaining bullet comparisons was 19.2%. Overall, twenty-two percent of all examiners made at least one false positive error. Roughly half of the other responses were Inconclusive, and the remaining third were objectively correct Eliminations.

**Table 1.** Summary of all responses for CTS Test 23–5262

	Item 2	Item 3	Item 4	Item 5
Yes (Identification)	57 (20.4%)	51 (18.2%)	1 (0.4%)	53 (18.9%)
No (Elimination)	88 (31.4%)	92 (32.9%)	275 (98.2%)	92 (32.9%)
Inconclusive	135 (48.2%)	137 (48.9%)	3 (1.1%)	135 (48.2%)

A 20% false positive error rate for any measurement and classification tool—whether it be a license plate reader at a toll booth or a defective cookie detector on an assembly line—would be inconvenient, at best, and may pose considerable fiscal or security risks, at worst. Indeed, the Food and Drug Administration has stopped the use of life-saving medical devices based on a .022% chance of death—1,000 times less than the false positive rate reported here (14). For forensic evidence presented in a court of law, however, a 20% false-positive error rate ups the ante by increasing the risk of wrongful conviction and allowing guilty perpetrators to evade punishment.

## What Accounts for Poor Examiner Performance?

In response to concerns raised by these findings, AFTE convened a committee to investigate the unusually high error rate on CTS test 23-5262 (15). The committee published its findings on November 25, 2024, concluding that these errors were not the result of swapped samples or other quality control issues—they were bona fide errors in the examiner’s decision.

The AFTE committee gathered feedback from several examiners who had made false positive errors to better understand their decision-making processes. The findings revealed no singular cause but highlighted recurring themes:

1. External Influences on Conclusions: Some examiners allowed task-irrelevant information or expectations to influence their identification conclusions, rather than basing decisions solely on evidence analysis.
2. Policy Misunderstandings: Some examiners misunderstood their laboratory’s policy on inconclusive results, leading them to “guess.”
3. Misjudging Agreement (similarity of compared items): Some examiners conflated a lack of disagreement with sufficient agreement, resulting in incorrect identifications.

The AFTE committee recommended several procedural and instructional changes, including refining CTS test methodologies and improving examiner education regarding laboratory policies (16).

AFTE’s proactive approach to understanding the root causes of these errors is commendable and provides valuable insights into both the CTS proficiency test regime and the broader practice of the forensic firearm discipline. We note, however, that the themes identified in the AFTE report (15) focus on examiners’ failures rather than on attributes of test design and administration. As a result, the AFTE Report overlooks or minimizes several foundational issues within the firearm and toolmark discipline. These overlooked factors, both structural and procedural, present serious challenges to the field’s credibility and effectiveness. The purpose of this article is to identify and explore these critical factors. We offer suggestions to improve both the practice and its testing.

Before delving into the specific factors that challenge this discipline, it is useful to consider attributes of a proficiency test that would make it useful in this context.

## What Should We Expect from a Proficiency Test?

Proficiency tests are employed in a variety of domains, such as medical practice, pipefitting, tugboat piloting, archery, and accounting, to assess the accuracy of performance for specific aptitudes or skills in situ. Results commonly serve as an objective basis for developing training programs, guiding work assignments, and predicting performance accuracy (i.e., validating) when applying aptitudes or skills outside the testing context (i.e., in the real world). To these ends, the following attributes are critical.

At the outset, we note that there is nothing particularly novel about these attributes—their importance is widely recognized in many academic and applied discussions of performance evaluation (17)—but they deserve greater attention in the forensic context (18). We separate these attributes into test design and test administration.

**Test Design Attribute**
  1) *Proficiency tests must be capable of differentiating between the best and worst performers.* Human performance on perceptual or cognitive tasks naturally varies among individuals and over time, owing in part to the differential sensitivity of sensory measurement, memory, and decision criteria employed for classification. Performance tests can capture this variation only if they are designed to reveal variability in the data, i.e., so that test items manifest a range of difficulties. If a substantial proportion of the queries are answered either correctly or incorrectly, the collected data will exhibit ceiling or floor effects, making it difficult, if not impossible, to determine the performance distribution, including the true error rate and its relationship to task difficulty (19, 20).
  2) *Answers to all test queries must be scored.* Proficiency tests should have queries that vary in difficulty, and all answers—including inconclusive responses—must be used to compute accuracy. Selective scoring as a function of difficulty (e.g., omitting inconclusive responses) will bias determinations of proficiency.
  3) *Proficiency tests must define and justify their scoring rules—especially regarding how inconclusive responses are evaluated and whether correctness is anchored to ground truth or consensus.* Using an “absolute scoring” rule, accuracy is defined strictly as the fraction of queries answered correctly with respect to ground truth. By contrast, “consensus scoring” evaluates individual performance relative to the aggregated performance of the group. Either approach may be useful, depending on the context and nature of the test, but the scoring rule must be specified and justifiable.
  4) *Different instances of a proficiency test for the same ability must include queries of comparable clarity and difficulty.* This is important for fairness, of course. Use of consensus scoring (see above) is a common practice to compensate for tests that differ in difficulty. But the stability of test material is essential if absolute scoring is used to make meaningful predictions of performance accuracy in the real world.
**Test Administration Attributes**
  5) *Task-irrelevant information should be excluded from the testing program.* The purpose of a proficiency test is to determine the ability of an individual to solve a specific type of problem: Is it red or green? Is it bigger than a breadbox? Contextual information, including such things as knowledge of the probability of occurrence of certain test items or the inclusion of a “none of the above” option in a multiple-choice test, can influence expectations and significantly alter the decision criteria an individual employs for classification (21, 22).
  6) *Nonblind verification and other unvalidated practices should not be used*. Verification is intended to serve as an independent check on examiner conclusions, but when the second examiner is aware of the first examiner’s decision, the process becomes contaminated. Rather than catching errors, nonblind verification tends to reinforce them, creating a feedback loop that falsely suggests corroboration. Despite its widespread use, nonblind verification lacks a scientific foundation and should be abandoned. If verification is to be employed, it must be conducted in a blind manner, and additional research is urgently needed to determine its actual effectiveness.

## How Do CTS Proficiency Tests Conform to These Ideals and What Does that Reveal About Forensic Practice?

### On Test Difficulty: Easy Out of Class Eliminations.

#### Proficiency tests must be capable of differentiating between the best and worst performers.

CTS proficiency tests routinely require comparisons of test items that differ in class characteristics. Class characteristics are objective and measurable features shared across firearms of the same design, such as caliber or the number and direction of lands and grooves in the gun barrel. Eliminations based on class characteristics are considered easy, given that the comparisons involve no subjective interpretation (13). One might wonder about the value of including such comparisons on a proficiency exam; would we ask what a red stoplight signifies in a professional driver’s exam? And what inferences could be drawn if such easy questions are answered correctly? While such questions may identify individuals who lack even minimal task competence, correct responses do not meaningfully differentiate proficiency among trained examiners.

Indeed, inclusion of easy test queries naturally skews the distribution of responses to the ceiling—98% of examiners tested in CTS 23-5262 correctly called the out-of-class query an elimination—which is largely useless information for differentiating the range of examiner performance. Nonetheless, one glimmer of insight is revealed by these queries: despite the general ease of out-of-class comparisons, performance was not 100%. Surprisingly, 1.5% of examiners in the CTS study did not call the out-of-class comparison an elimination. One inexplicably called it an Identification, and four examiners called it Inconclusive.

This is not the only known instance where examiners were confused by class characteristics. A large-scale validation study reportedly had no out-of-class comparisons by design; yet, approximately one-third of all eliminations were reportedly based on class characteristics (23). Either the study design was misdescribed and contained easy out-of-class eliminations, or participating examiners were confused about what constitutes a class characteristic. In another study, numerous examiners reported an out-of-class comparison as inconclusive (24). The 2024 Rhode Island crime lab incident provides another example of examiners failing to understand class characteristics, this time in actual casework (25). In that case, three examiners concluded that an out-of-class comparison was an Identification—an obvious impossibility.

Understanding how this subset of examiners failed an easy query could help improve forensic practice. The authors of the AFTE Report, however, did not find that the inconclusive responses for out-of-class comparisons were errors. Rather, the Report (15) states:

Assuming the bullets in these test kits exhibited *discernible* class characteristic differences (i.e., polygonal rifling vs. conventional rifling), then these inconclusive responses would not be appropriate. Discernible class characteristic differences provide compelling evidence of two different firearms. (emphasis in original, p. 20)

To be clear, these examiners indicated they could not distinguish between polygonal rifling (Item 4) and conventional rifling (Item 1), and the Report asserts this is not an error if the difference between polygonal and conventional rifling is not “discernible.”

The AFTE glossary uses the word “discernible” but does not define it (26). In perceptual science discernability refers to the ability of an observer to perceive differences between stimuli.^‡^ Lack of discernibility is, according to the AFTE Report, not an error but cause for an inconclusive assignment. The important question here, however, is not whether the examiners erred, but why they did so. Why did they fail to distinguish patterns that were readily discriminable by 275 of their cohorts and are famously easy to classify correctly? These circumstances point to a failure of practice—to a failure of the examiner’s visual sensitivity, attentional focus, perceptual fidelity, and/or visual cognition—not to any particular property of the patterns themselves. In other words, the examiners in question were not sufficiently trained on this type of problem to apply their visual measurement and classification skills correctly.

The inclusion of out-of-class eliminations on the CTS exam is, on the one hand, troubling in that it provides an easy comparison that reveals little about individual proficiency or the distribution of performance within the tested group. On the other hand, the fact that *any* examiner did not answer this comparison correctly is concerning and suggests fundamental deficiencies in training. While training to become a firearm examiner focuses on individual (“unique”) characteristics on fired bullets or cartridge cases, these findings suggest that more basic training on class characteristics may be needed.

Finally, these questions of task difficulty raise a broader concern about whether proficiency tests correspond to the difficulty of comparisons encountered in actual casework. As implemented today, proficiency tests “are typically designed to be relatively easy for a competent analyst to pass” (9)—suggesting that they may not be comparable to casework or designed with that intent. The results of proficiency tests are, nonetheless, frequently cited in legal proceedings. We see this both as an egregious distortion of evidence and a missed opportunity to properly validate examiner performance. In particular, error rates are sensitive to the difficulty of the comparisons presented, and without evidence showing that CTS test materials reflect the distribution of difficulty encountered in real casework, the resulting false-positive rates cannot be assumed to represent the risk of error in practice, which is what a court would want to know. Furthermore, because these results do not meaningfully differentiate among examiners with more than a minimal level of competence, they are of limited utility for laboratory identification of relevant training needs and for channeling work assignments beyond that level. The recommendations we make herein are intended to overcome these problems by capitalizing on opportunities for validation through well-designed proficiency tests.

### The Issue of Inconclusive Results: A Missing Explanation and Unproductive Commentary.

#### All answers to test queries must be scored.

In addition to definitive “identification” and “elimination” decisions, forensic examiners may report that a given pattern comparison is inconclusive. This latter option is a reasonable response in real casework where fired bullets might be degraded by environmental or other factors that affect the ability to reach a definitive conclusion. As many have noted, however, it complicates efforts to assess examiner performance (27).

Almost half of the responses in the CTS Test 23-5262 were Inconclusive. How should these responses be treated, and what do they mean for efforts to understand proficiency? The AFTE Report (15) suggests that Inconclusive responses can sometimes be incorrect:

The committee wants to be clear that inconclusive results should not always be accepted without question. Laboratories must evaluate the inconclusive results and determine whether or not they are appropriate given the condition of the test materials and in consideration of laboratory protocols. If determined not to be appropriate, corrective action should be taken. (p. 21).

However, the Report fails to explain how and when an inconclusive result is not appropriate. Is the claim that inconclusive is wrong because the test materials, in hindsight, were adequate to support a definitive conclusion? If so, the Report offers no criteria for determining this adequacy. Without further clarification, the Report’s guidance on this issue is incomplete and unhelpful.

The Report further highlights a troubling issue: Some examiners do not understand their laboratory policies regarding the use of the inconclusive category. For instance, one examiner “believed inconclusive results were no longer allowed after a change to their quality assurance manual” [(15) p. 21]. This misunderstanding led the examiner to report a definitive conclusion they were “nearest to,” rather than the inconclusive result they initially planned to submit. This incident occurred at an accredited laboratory, raising serious concerns about the effectiveness of lab policies in communication and understanding. This confusion directly contributes to errors, as examiners feel pressured to conform to perceived expectations rather than follow appropriate forensic practices.

This is not the first evidence that practicing examiners do not fully comprehend and/or misapply the Inconclusive category. For example, after analyzing the data from a well-known study of firearm examiners, the authors concluded:

The language used to define the AFTE Range of Conclusions is specific and concise, but our data [from the Ames I study] suggest that interpretation and application of this system is not consistent across the profession. Rather, our analysis strongly suggests that, as currently applied, the categories of the current five-point AFTE Range of Conclusions scale cannot be said to have consistent, meaningful interpretations. One interpretation of these results is that the use of a five-point scale should at minimum be implemented with a more coordinated training program (p. 4) (28).

Dror and Scurich’s initial observation that inconclusives can be a correct or incorrect decision sparked a heated debate in the forensic literature (29). Some commentators have taken an absolutist stance, insisting that inconclusive results are never “errors” because they do not make a claim about the ground-truth of the two items (30, 31).^§^ Historically, AFTE also never considered an inconclusive judgment to be erroneous (13). Fortunately, the Report does not argue that Inconclusives are never errors, which encourages efforts to critically evaluate the appropriateness of examiner decisions, including inconclusive ones. For example, when an inconclusive result is reached in a situation where the test materials clearly support a definitive identification or elimination, it could indicate a lack of examiner confidence, inadequate training, or even cognitive bias.

At the same time, the AFTE Report critiques inconclusive results without offering actionable solutions or guidelines, leaving examiners uncertain about when such outcomes might be deemed inappropriate. To address this issue productively, the forensic community needs clear, evidence-based criteria for evaluating the appropriateness of inconclusive results (32). For example, if inconclusive outcomes are deemed problematic when test materials are adequate to support a definitive conclusion, a transparent and consistent standard must be established for assessing material quality. Additionally, laboratories should implement training programs to help examiners distinguish legitimate uncertainty from unwarranted hesitation when assessing the sufficiency of evidence. Laboratories must ensure that their policies on inconclusive results are explicitly communicated and understood by all examiners, especially in accredited labs where such misunderstandings should not occur.

Finally, we note that there is a scientifically grounded alternative to the AFTE inconclusive option that addresses the “discernability” issue raised in the AFTE Report and circumvents awkward questions about the appropriateness (and meaning) of inconclusive results. This alternative (reviewed in ref. 33), which is rooted in Signal Detection Theory (34), sheds greater light on examiner performance—particularly the murky depths of inconclusive decisions—by acquiring examiner confidence ratings for all judgments. These ratings can be used to estimate continuous variation in the accuracy of decisions and to compute a measure of discriminability for each examiner, all of which provides a richer testament to an examiner’s proficiency. We recommend that the discipline move in this direction.

### The Role and Limitations of Consensus in Proficiency Testing.

#### Proficiency tests must define and justify their scoring rules: Especially how inconclusive responses are evaluated and whether correctness is anchored to ground truth or consensus.

CTS does not exclusively use ground truth to determine the correct answers on its tests. It also uses the consensus response to determine the “expected” answer. Consensus scoring offers a practical approach to proficiency testing in a field where there are no clear, quantifiable criteria for determining whether toolmarks are in “sufficient agreement” for a definitive conclusion. It serves as a proxy for objectivity by drawing on the collective expertise of examiners (35). Majority agreement provides some confidence in the reasonableness of a decision, even in the absence of fixed sufficiency standards. This method helps validate proficiency by assessing how closely individual results align with the broader forensic community, aiding in the identification of outliers and systemic issues. Researchers sometimes use the term “forensically correct response” when employing consensus scoring (36).

In the case of the 23-5262 CTS test, Elimination was deemed a correct response because it reflected the ground truth; however, Inconclusive was also scored as correct because it was the most common response.

The AFTE Report takes a contradictory position on consensus scoring. While it relies on consensus to define the “expected” results for the proficiency test, the Report (15) argues that “the use of test consensus to judge the ‘correctness’ of an individual’s responses may be inappropriate” (p. 17). It discusses a hypothetical scenario in which the consensus response contradicts the ground truth, so a response consistent with the ground truth—but not the consensus—would be deemed incorrect, despite being “objectively correct.”^¶^ However, if being “objectively correct” is the criterion, then any nonelimination response on the 23-5262 CTS test is wrong because Item 1 and Items 2, 3, 4, and 5 were not fired by the same gun. In other words, all the inconclusive responses are errors.

Reliance on consensus introduces several challenges. First, consensus scoring assumes that the majority is correct. This can, in theory, lead to systemic errors if the majority’s judgment is flawed. Second, consensus scoring can, in theory, penalize highly skilled examiners. As the Report (15) notes, “a particularly skilled examiner might pick up on one or more subtle cues... [and be] scored as an error even when it is objectively correct” (p. 17).

Regarding the first concern, it is fundamentally a definitional issue. If consensus scoring is adopted as the criterion for correctness, one cannot simultaneously argue that the consensus response is “wrong.” As for the second concern, there is no empirical method for distinguishing “skilled” examiners from unskilled ones, rendering this argument moot (37). Note that some commentators advocate for excluding inconclusive results from error rate calculations; doing so would significantly increase the false positive error rates to as high as 35% on CTS test 23-5262.

The AFTE Report laments that “the consensus result cannot even be determined until after test participants submit their results and the results are tabulated” (p. 21). This is not correct. Consensus scoring does not require post hoc analyses of responses. Prescreening can be used to determine the correct response for a given test comparison. In fact, this occurred with CTS test 23-5262. The host laboratory that created the test exemplars had “multiple AFTE certified examiners” (p. 3) who prescreened and verified the test items. Then, three laboratories conducted “predistribution testing,” and based on those results, CTS determined that both elimination and inconclusive responses were correct. Hicklin et al. (38) similarly had a panel of experts evaluate the “quality” of “case-like distorted and fragmented bullets” (p. 4) prior to conducting their error-rate study. Both are examples of consensus scoring that was determined ex ante.

While consensus scoring is useful in fields without clear objective standards, such as art appraisals and judging figure skating competitions, the forensic community should strive to reduce its reliance on this approach. Emerging technologies, such as 3D surface analysis and automated scoring systems, offer promising alternatives (32). These tools can quantify toolmark similarity, which can be used to set normative thresholds for inconclusives. The AFTE Report analyzed a nonrandom sample of the CTS test sets to assess the “geometric surface similarity” of the test items. Two critical findings emerged. First, there was significant variability in the markings left on bullets even when fired from the same gun. This adds yet another layer of complexity to the evaluation of firearm examiner decisions, as discussed next. Second, most similarity scores were exceedingly low in interitem comparisons, yet the examiners’ modal response was inconclusive, raising the question of what threshold for elimination should be and whether such responses are incorrect (39).

### The Reproducibility of Judgments *and* Test Items.

#### Everybody should get the same kind of test.

One of the biggest revelations of the AFTE Report is the significant set-to-set variation across the tests, despite the bullets being fired from the same guns. As noted in the Report (15),

One member of the PTRC purchased two test sets for their laboratory. The bullets in one test set were adequately marked such that it was determined definitive conclusions were appropriate. In the second test set the bullets were not adequately marked and conclusive results could not be supported (i.e., inconclusive results were the most appropriate response). (p. 15).

Setting aside the issue of one person deciding which responses are appropriate, this observation introduces a significant confound in the test results: differences in responses could be due to examiner skill or test materials. The implication is that pooling responses across examiners is inappropriate. In other words, the examiners did not complete the ‘same’ test, so combining results from different tests does not make sense. Since each test is different, each one needs to be scored individually based on the quantity and quality of the available information. Exactly who should do this and how remains unclear.

Shot-to-shot variability has already been noted in the literature. To overcome this issue, Law and Morris created “double cast” plastic cartridge cases, which reproduce the original cartridge case almost exactly (40). This allowed examiners to compare the ‘same’ cartridge cases. Even when comparing the same cartridge cases, there was significant variability in the responses, which, by implication, is attributable to differences in examiners’ judgments rather than variance in test materials.

Aside from creating problems with aggregating test data, significant shot-to-shot variability poses a serious threat to the fundamental premise of firearm identification: that each firearm imparts unique characteristics that are reproducible. Without reproducibility, it is unclear whether the observed marks are truly linked to the firearm or are random, transient imperfections caused by external factors, such as variability in ammunition (e.g., casing material or bullet composition) or environmental conditions (e.g., residue buildup, lubrication, or barrel corrosion). If firearm-generated marks are not sufficiently reproducible across firing events, the basis for attributing those marks to a particular firearm becomes uncertain and potentially untenable.

The challenge becomes even greater in situations where two bullets are recovered, but no firearm is—a scenario that is not uncommon in casework. Without the firearm, there is no way to test whether individual characteristics reproduce consistently. This raises a critical question: How can an examiner determine whether a given mark is meaningful or merely incidental? In the absence of predictable or reproducible marks, the comparison process devolves into searching for similarities between the markings on the two bullets. This approach is highly prone to confirmation bias (discussed in the next section). However, even if similarities are found, they provide little information, as the examiner has no means to determine whether the markings are unique to the missing firearm or are subclass characteristics shared by a subset of firearms. Without a firearm to confirm the reproducibility of marks, examiners should refrain from drawing conclusions about individual characteristics, as there is no reliable way to distinguish individual marks from subclass marks. At most, examiners can comment on class characteristics when a firearm is unavailable for examination

### Cognitive Bias and the Power of Expectation.

#### Task-irrelevant information should be excluded from the testing program.

The influence of expectations on behavior in scientific research is well known, highlighting the need for methods such as blind and double-blind studies (41, 42). These methods aim to reduce expectation biases by ensuring participants, and sometimes researchers, do not know key details such as whether a participant received an active drug or a placebo. For example, in clinical trials, knowing they received a placebo can unintentionally affect participants’ reported outcomes or adherence to treatment plans. A similar effect has been observed in medical diagnostics, particularly in radiology, where cognitive biases can affect diagnostic accuracy. For instance, radiologists sometimes interpret imaging studies based on their expectations, which can lead to mistakes when those expectations are incorrect (43).

It has long been recognized that firearm identification tests often contain suggestive elements that can unintentionally influence participants’ judgments. One well-known firearm examiner study noted that “the participants were not told whether the questioned casings constituted an open or closed set. However, from the questionnaire/answer sheet, participants could have assumed it was a closed set and that every questioned casing should be associated with one of the ten slides” (44, p. 18). This methodological detail created a suggestive testing environment by implying that all bullets should have a match, effectively ruling out the possibility of eliminations (45). A different test with an unusually high false positive error rate made the same observation (46):

There was a test-taking bias, with examiners expecting each questioned sample to match at least one of the exemplars. This was definitely the case, as at least one examiner with over 30 years of experience admitted that he/she assumed all questioned samples linked back to one of the known specimens. (p. 129).

This also seemed to have occurred during this CTS test, in which participants were “assuming an identification had to be present” [(15) p. 20]. Although some participants reported that this expectation caused them to make “their best guess,” the expectation of at least one match probably led others to perceive enough agreement even if the items were not fired by the same gun.

The expectations to find a match in casework are potentially even stronger. Investigators often provide contextual information about the case (e.g., a known suspect’s firearm is believed to have been used), which can create an implicit expectation of a match. Indeed, some case submission forms are quite explicit that the questioned item originates from a specific source (e.g., “Please match these bullets to this gun”), thereby encouraging the expectation of a match. High caseloads and tight deadlines can lead examiners to assume matches rather than exhaustively exploring the possibility of no match or elimination (47).

Perhaps the most suggestive is an investigative lead generated through the National Integrated Ballistic Information Network (NIBIN) (48). NIBIN uses the Integrated Ballistic Identification System (IBIS) technology to compare and link digital images of cartridge casings and bullets recovered from crime scenes or test-fired firearms. When a potential connection between ballistic evidence from different incidents is identified, it is referred to as a NIBIN lead. The NIBIN output explicitly states that the “lead must be confirmed by a trained firearms examiner through traditional microscopic comparison.” The lead literally instructs an examiner to confirm the match. It is difficult to imagine anything more suggestive.

### Nonblind Verification in Forensic Science: Worse than Ineffective.

### Nonblind verification and other unvalidated practices should not be used.

One of the test participants from CTS 23-5262 who committed a false positive noted that his “conclusions were verified independently, but not blinded, by another qualified examiner from the same laboratory and the verifier agreed with [the examiner’s] conclusions without any further discussion” (15, p. 9). The critical point is that the verification was “not blind.” Instead of providing an independent check, nonblind verification reinforces errors, creating a feedback loop that confirms them rather than correcting them. Moreover, nonblind verification is worse than ineffective to the extent that it gives the impression that additional examiners independently reached the same conclusion.

There is only a single empirical study on the impact of verification in firearm examinations. The results differ significantly depending on whether the verifier was blind to the first examiner’s conclusion (49). The odds of disagreement in conclusions were five times higher when the second examiner was blind than when the examiner was not blind (i.e., was aware of the first examiner’s conclusion). This effect was more pronounced when the first examiner held a higher professional status (e.g., permitted to testify in court) than the second examiner, providing clear evidence of bias. Notably, this European study examined casework in which the ground truth was unknown, and thus it did not assess whether verification improves accuracy or detects errors.

There is simply no legitimate reason that verification—if it is to be done at all—should be nonblind. Misleading hedges like “independent verification” should be jettisoned from the forensic lexicon. Additional empirical studies are critically needed to evaluate the efficacy of verification (50). The issue is not simply whether verification is effective, but the extent to which it is and under what conditions.

## Conclusion

The results from the 2023 CTS proficiency test have significant implications for the criminal justice system, especially regarding the admissibility, interpretation, and significance of forensic firearm evidence in court (51). A false positive error rate nearing 20%—and even higher rates under different scoring assumptions—undermines the long-held claim that firearm examiners make highly accurate identifications.^#^ At a minimum, these findings call for heightened judicial scrutiny of expert qualifications and the scientific foundations of firearm identification pursuant to admissibility standards such as *Daubert* (52) and *Frye* (53). Judges should be cautious about claims of near-perfect accuracy, especially when they come from simple proficiency tests. Although proficiency testing has great potential for forensic practice (9, 10), evidence indicates that many current proficiency testing practices conceal fundamental weaknesses in examiner performance and test design, undermining the utility of test results for laboratory quality control, accreditation, and predicting performance accuracy.

The systemic issues exposed by consideration of CTS 23-5262—ranging from the use of tests that are not representative of real casework, to inadequate training in basic class characteristics, to nonblind verification practices, to the cognitive influence of suggestive investigative leads—highlight how courtroom testimony may be shaped by forces far removed from objective, reproducible science. These structural flaws pose serious risks of wrongful conviction, especially when firearm identifications are presented as definitive and unassailable (54). While AFTE’s proactive approach to understanding the root causes of these problems is commendable, the issues must be addressed through systemic reform. Continued reliance on overstated forensic certainty threatens to erode public trust in the criminal justice system and perpetuate unjust outcomes. The path forward requires not only technical refinement but also a cultural shift toward transparency, scientific rigor, and epistemic humility in the use of forensic evidence. This path can be achieved by promoting awareness, vigilance, and candor among all parties and by fostering collaboration among forensic practitioners, police, legal professionals, and the broader scientific community.

## Footnotes

^*^In practice, forensic observers typically compare two or more patterned impressions. Assertions of identity refer to the hypothesis that the patterns were produced by the same physical object or process.

^†^“[Forensic proficiency] tests can serve many purposes including but not limited to training and testing personnel, improving laboratory practices and procedures, and identifying future needs for a laboratory. Properly designed, proficiency tests may also provide a reasonable estimate of the rate at which false discoveries, false positive errors, and false negative errors occur” (1 at p. 1089).

^‡^What actually matters in forensic pattern comparison is not whether an examiner can *perceive* the difference between patterns, but rather whether they can *discriminate* between them. “Discernible” is thus a curious choice. It appears to reflect a belief that if an examiner is not consciously aware of—i.e., cannot perceive—a difference between patterns, then it makes no sense to try to discriminate between them. Hence, the examiner defaults, the comparison is deemed inconclusive, and that decision is not recorded as an error. This logic betrays a misunderstanding of how visual pattern classification works. As others have argued (e.g., 33), discrimination of forensic patterns need not be limited to those that are discernible. There are distinct advantages to avoiding that limitation, including a reduction of inconclusive judgments.

^§^Much of the commentary on this issue has been semantic rather than substantive. For example, some argue that inconclusives can be “inappropriate” but never “incorrect” or “errors” (55). These meaningless distinctions lead to absurd conclusions. For example, that inconclusives are “neither correct nor incorrect” (56). These semantic maneuvers do nothing to clarify how such decisions should be evaluated empirically or how they affect the accuracy and probative value of forensic conclusions, which are the issues that matter for science and the law.

^¶^The opposite pattern was once observed in a proficiency test of forensic document examiners: 100% of examiners gave incorrect responses from a ground truth perspective, though they were all “correct” from a consensus perspective. In other words, perfect reliability, zero validity (see 57).

^#^There is a substantial literature arguing that forensic experts should testify about the likelihood ratio (the conventional “weight of evidence”) (58). The CTS test in question cannot measure the true positive rate (TRP)—the numerator of the likelihood ratio—because it did not include any same-source comparisons; it can only measure the false positive rate (FPR)—the denominator—since all of the comparisons were different sources. Under a generous assumption that the TPR is close to 1.0, the LR would be no greater than 1.0/0.20 = 5. According to the Association of Forensic Science Providers (59), this is considered “weak” evidence—certainly much more so than is claimed by firearm examiners testifying that the likelihood of a different gun fired the munition is a “practical impossibility” or that an Identification indicates “extremely strong support for common source” (60). We also note that Aggadi et al. (61) applied a complex (ordered probit) modeling technique to the well-known large-scale validation study (Ames II) and found “the likelihood ratios can be as low as less than 10”.

## References

[1] J. J. Koehler, Fingerprint error rates and proficiency tests: What they are and why they matter. Hastings LJ **59**, 1077 (2008).

[2] National Research Council, Strengthening Forensic Science in the United States: A Path Forward (National Academies Press, Washington, DC, 2009).

[3] K. L. Monson, E. D. Smith, S. J. Bajic, Planning, design and logistics of a decision analysis study: The FBI/Ames study involving forensic firearms examiners. Forensic Sci. Int. Synergy **4**, 100221 (2022). 35243285 10.1016/j.fsisyn.2022.100221 PMC8860930

[4] D. L. Faigman, N. Scurich, T. Albright, The field of firearms identification is flawed. Scientific American (2022). https://www.scientificamerican.com/article/the-field-of-firearms-forensics-is-flawed/. Accessed 11 June 2026.

[5] J. J. Koehler, Forensics or fauxrensics? Ascertaining accuracy in the forensic sciences. Ariz. State Law J. **49**, 1369–1414 (2017).

[6] Collaborative Testing Services, Forensics testing overview. https://cts-forensics.com/index-forensics-testing.php. Accessed 11 June 2026.

[7] Collaborative Testing Services, Program 3: Proficiency testing in firearms. https://cts-forensics.com/program-3.php. Accessed 11 June 2026.

[8] R. G. Nichols, Defending the scientific foundations of the firearms and tool mark identification discipline: Responding to recent challenges. J. Forensic Sci. **52**, 586–594 (2007). 17456086 10.1111/j.1556-4029.2007.00422.x

[9] US Department of Justice, National Commission on Forensic Science, Performance in crime laboratories through testing and feedback (Publication No. NCFS 864776) (2016). https://perma.cc/G6XV-P72E. Accessed 11 June 2026.

[10] B. L. Garrett, G. Mitchell, The proficiency of experts. Univ. Pa Law Rev. **166**, 901–968 (2017).

[11] Collaborative Testing Services, CTS statement on the use of proficiency testing data for error rate determinations (2010).

[12] Collaborative Testing Services, Report 23–5262 (2023). https://cts-forensics.com/reports/23-5262_Web.pdf. Accessed 11 June 2026.

[13] M. A. Keisler, S. Hartman, A. Kilmon, M. Oberg, M. Templeton, Isolated pairs research study. AFTE J. **50**, 56–58 (2018).

[14] Reuters, FDA classifies recall of Medtronic embolization devices as most serious (2025). https://www.reuters.com/business/healthcare-pharmaceuticals/fda-classifies-recall-medtronic-embolization-devices-most-serious-2025-03-18/. Accessed 11 June 2026.

[15] Association of Firearm and Tool Mark Examiners, PTRC final report (with Erratum) (2025). https://afte.org/wp-content/uploads/2025/03/PTRC_Final_Report_2024-11-25_with_Erratum.pdf. Accessed 11 June 2026.

[16] Association of Firearm and Tool Mark Examiners, CTS PTRC statement (2025). https://afte.org/wp-content/uploads/2025/03/CTS_PTRC_Statement.pdf. Accessed 11 June 2026.

[17] N. Scurich, D. L. Faigman, T. Albright, Scientific guidelines for evaluating the validity of forensic feature-comparison methods. Proc. Natl. Acad. Sci. U.S.A. **120**, e2301843120 (2023). 37782809 10.1073/pnas.2301843120 PMC10576079

[18] J. J. Koehler, Proficiency tests to estimate error rates in the forensic sciences. Law Probab. Risk **12**, 89–98 (2013).

[19] R. E. Gutierrez, More unjustified inferences from limited data in Guyll et al. (2023). Law Probab. Risk **23**, mgae014 (2024).

[20] R. E. Gutierrez, E. J. Prokesch, The false promise of firearms examination validation studies: Lay controls, simplistic comparisons, and the failure to soundly measure misidentification rates. J. Forensic Sci. **69**, 1334–1349 (2024). 38684627 10.1111/1556-4029.15531

[21] G. Edmond , Thinking forensics: Cognitive science for forensic practitioners. Sci. Justice **57**, 144–154 (2017). 28284440 10.1016/j.scijus.2016.11.005

[22] W. C. Thompson, Shifting decision thresholds can undermine the probative value and legal utility of forensic pattern-matching evidence. Proc. Natl. Acad. Sci. U.S.A. **120**, e2301844120 (2023). 37782790 10.1073/pnas.2301844120 PMC10576151

[23] N. Scurich, H. Stern, Commentary on: Monson KL, Smith ED, Peters EM. Accuracy of comparison decisions by forensic firearms examiners. J. Forensic Sci. **68**, 1093–1094 (2023). 37083238 10.1111/1556-4029.15258

[24] B. A. Best, E. A. Gardner, An assessment of the foundational validity of firearms identification using ten consecutively button-rifled barrels. AFTE J. **54**, 28–37 (2022).

[25] N. Scurich, Firearm forensics is still troubled by systemic failure. Scientific American (2025). https://www.scientificamerican.com/article/firearm-forensics-is-still-troubled-by-systemic-failure/. Accessed 11 June 2026.

[26] Association of Firearm and Tool Mark Examiners, AFTE glossary (Version 6.091922) (2024). https://afte.org/wp-content/uploads/2024/11/AFTE_Glossary_Version_6.091922_FINAL_COPYRIGHT.pdf. Accessed 11 June 2026.

[27] N. Scurich, R. S. John, Three-way ROCs for forensic decision making. Stat. Public Policy **10**, 2239306 (2023).

[28] D. P. Baldwin, S. J. Bajic, M. D. Morris, D. S. Zamzow, A study of examiner accuracy in cartridge case comparisons. Part 2: Examiner use of the AFTE range of conclusions. Forensic Sci. Int. **349**, 111739 (2023). 37257389 10.1016/j.forsciint.2023.111739

[29] I. E. Dror, N. Scurich, (Mis)use of scientific measurement in forensic science. Forensic Sci. Int. Synergy **2**, 333–338 (2020). 33385131 10.1016/j.fsisyn.2020.08.006 PMC7770438

[30] H. R. Arkes, J. J. Koehler, Inconclusives are not errors: A rejoinder to Dror. Law Probab. Risk **21**, 89–90 (2022).

[31] A. M. Smith, T. M. Neal, The distinction between discriminability and reliability in forensic science. Sci. Justice **61**, 319–331 (2021). 34172120 10.1016/j.scijus.2021.04.002

[32] M. Cuellar, S. Gao, H. Hofmann, An algorithm for forensic toolmark comparisons. Forensic Sci. Int. Synergy **9**, 100543 (2024). 39156234 10.1016/j.fsisyn.2024.100543 PMC11327548

[33] T. D. Albright, How to make better forensic decisions. Proc. Natl. Acad. Sci. U.S.A. **119**, e2206567119 (2022). 36099301 10.1073/pnas.2206567119 PMC9499562

[34] D. M. Green, J. A. Swets, Signal Detection Theory and Psychophysics (John Wiley & Sons Inc., New York, NY, 1966).

[35] D. J. Weiss, J. Shanteau, Empirical assessment of expertise. Hum. Factors **45**, 104–116 (2003). 12916584 10.1518/hfes.45.1.104.27233

[36] R. A. Hicklin , Accuracy and reliability of forensic handwriting comparisons. Proc. Natl. Acad. Sci. U.S.A. **119**, e2119944119 (2022). 35914157 10.1073/pnas.2119944119 PMC9371688

[37] R. E. Gutierrez, C. Addyman, Commentary on: Monson KL, Smith ED, Peters EM. Accuracy of comparison decisions by forensic firearms examiners. J. Forensic Sci. **68**, 86–100 (2023). 36183147 10.1111/1556-4029.15152 PMC10092368

[38] R. A. Hicklin , Accuracy and reproducibility of bullet comparison decisions by forensic examiners. Forensic Sci. Int. **365**, 112287 (2024). 39547116 10.1016/j.forsciint.2024.112287

[39] M. Cuellar, The problem with eliminations: Why forensic comparisons need false negative rates. Forensic Sci. Int. Synergy **11**, 100621 (2025). 40791577 10.1016/j.fsisyn.2025.100621 PMC12337876

[40] E. F. Law, K. B. Morris, Evaluating firearm examiner conclusion variability using cartridge case reproductions. J. Forensic Sci. **66**, 1704–1720 (2021). 34057735 10.1111/1556-4029.14758

[41] D. M. Risinger, M. J. Saks, W. C. Thompson, R. Rosenthal, The Daubert/Kumho implications of observer effects in forensic science: Hidden problems of expectation and suggestion. Calif. Law Rev. **90**, 1–56 (2002).

[42] I. E. Dror, D. Charlton, A. E. Péron, Contextual information renders experts vulnerable to making erroneous identifications. Forensic Sci. Int. **156**, 74–78 (2006). 16325362 10.1016/j.forsciint.2005.10.017

[43] Ö. Önder , Errors, discrepancies and underlying bias in radiology with case examples: A pictorial review. Insights Imaging **12**, 51 (2021). 33877458 10.1186/s13244-021-00986-8 PMC8056102

[44] T. G. Fadul Jr., G. A. Hernandez, S. Stoiloff, S. Gulati, An empirical study to improve the scientific foundation of forensic firearm and tool mark identification utilizing 10 consecutively manufactured slides (Final Report No. 237960). US Department of Justice (2012). https://www.ojp.gov/pdffiles1/nij/grants/237960.pdf. Accessed 11 June 2026.

[45] A. Stroman, Empirically determined frequency of error in cartridge case examinations using a declared double-blind format. AFTE J. **46**, 157–175 (2014).

[46] R. Nichols, Firearm and Toolmark Identification: The Scientific Reliability of the Forensic Science Discipline (Academic Press, London, UK, 2018).

[47] S. R. Garten, IBIS BrassTRAX correlation performance and review practices. AFTE J. **51**, 37–46 (2019).

[48] J. Budden, ATF’s NIBIN program. Law Order **49**, 101–106 (2001). National Criminal Justice Reference Service (NCJ No. 192535).

[49] E. J. A. T. Mattijssen, C. L. M. Witteman, C. E. H. Berger, R. D. Stoel, Cognitive biases in the peer review of bullet and cartridge case comparison casework: A field study. Sci. Justice **60**, 337–346 (2020). 32650935 10.1016/j.scijus.2020.01.005

[50] K. N. Ballantyne, G. Edmond, B. Found, Peer review in forensic science. Forensic Sci. Int. **277**, 66–76 (2017). 28622536 10.1016/j.forsciint.2017.05.020

[51] B. L. Garrett, N. Scurich, E. Tucker, H. Bloom, Judging firearms evidence and the Rule 702 amendments. Judicature **107**, 41–49 (2023).

[52] Daubert v., Merrell Dow Pharmaceuticals Inc, 509 U.S. 579 (1993).

[53] Frye v. United States, 293 F. 1013 (D.C. Cir. 1923).

[54] R. Valerio, N. Bunn, Firearm forensics has proven reliable in the courtroom. And in the lab. Scientific American (2023). https://www.scientificamerican.com/article/firearm-forensics-has-proven-reliable-in-the-courtroom-and-in-the-lab/. Accessed 11 June 2026.

[55] H. Swofford , Inconclusive decisions and error rates in forensic science. Forensic Sci. Int. Synergy **8**, 100472 (2024). 38737990 10.1016/j.fsisyn.2024.100472 PMC11087963

[56] M. Guyll, S. Madon, Y. Yang, K. A. Burd, G. Wells, Validity of forensic cartridge-case comparisons. Proc. Natl. Acad. Sci. U.S.A. **120**, e2210428120 (2023). 37155908 10.1073/pnas.2210428120 PMC10193974

[57] D. M. Risinger, M. P. Denbeaux, M. J. Saks, Exorcism of ignorance as a proxy for rational knowledge: The lessons of handwriting identification expertise. Univ. Pa Law Rev. **137**, 731–792 (1989).

[58] C. Aitken, F. Taroni, S. Bozza, Evidence, probability and relative plausibility. Int. J. Evid. Proof **26**, 309–324 (2022). 36110308 10.1177/13657127221114508 PMC9465537

[59] Association of Forensic Science Providers, Standards for the formulation of evaluative forensic science expert opinion. Sci. Justice **49**, 161–164 (2009). 19839414 10.1016/j.scijus.2009.07.004

[60] Organization of Scientific Area Committees for Forensic Science (OSAC), Standard scale of source conclusions and criteria for toolmark examinations (2019). https://www.nist.gov/system/files/documents/2020/03/24/100_fatm_roc_and_criteria_standard_asb_mar2019_OSAC%20Proposed.pdf. Accessed 11 June 2026.

[61] N. Aggadi, K. Zeller, T. Busey, Quantifying the strength of firearms comparisons based on error rate studies. J. Forensic Sci. **70**, 84–97 (2025). 39474778 10.1111/1556-4029.15646 PMC11693517

